# Genome-Wide Detection of Selective Signatures in Chicken through High Density SNPs

**DOI:** 10.1371/journal.pone.0166146

**Published:** 2016-11-07

**Authors:** Zhuang Liu, Congjiao Sun, Liang Qu, Kehua Wang, Ning Yang

**Affiliations:** 1 National Engineering Laboratory for Animal Breeding and MOA Key Laboratory of Animal Genetics and Breeding, College of Animal Science and Technology, China Agricultural University, Beijing, 100193, China; 2 Jiangsu Institute of Poultry Science, Yangzhou, Jiangsu, 225125, China; Estonian Biocentre, ESTONIA

## Abstract

Chicken is recognized as an excellent model for studies of genetic mechanism of phenotypic and genomic evolution, with large effective population size and strong human-driven selection. In the present study, we performed Extended Haplotype Homozygosity (EHH) tests to identify significant core regions employing 600K SNP Chicken chip in an F2 population of 1,534 hens, which was derived from reciprocal crosses between White Leghorn and Dongxiang chicken. Results indicated that a total of 49,151 core regions with an average length of 9.79 Kb were identified, which occupied approximately 52.15% of genome across all autosomes, and 806 significant core regions attracted us mostly. Genes in candidate regions may experience positive selection and were considered to have possible influence on beneficial economic traits. A panel of genes including *AASDHPPT*, *GDPD5*, *PAR3*, *SOX6*, *GPC1* and a signal pathway of *AKT1* were detected with the most extreme P-values. Further enrichment analyses indicated that these genes were associated with immune function, sensory organ development and neurogenesis, and may have experienced positive selection in chicken. Moreover, some of core regions exactly overlapped with genes excavated in our previous GWAS, suggesting that these genes have undergone positive selection may affect egg production. Findings in our study could draw a comparatively integrate genome-wide map of selection signature in the chicken genome, and would be worthy for explicating the genetic mechanisms of phenotypic diversity in poultry breeding.

## Background

The chicken have gone through intensive selection because of domestication and breeding, what has gave rise to various phenotypes when compared with their wild counterparts [1]. Selection signatures are the selective footprints across the organism genome due to the effect of artificial selection, which displayed the long range linkage disequilibrium in chromosome or genetic diversity reduction [2, 3]. Thus, identifying selection signatures in chicken, we could effectively and efficiently uncovered the selected genes and genomic regions, which would contribute to understand the relationships between genotype and phenotype.

Recently, with the development and application of high throughput and cost-effective genotyping techniques, the power of detecting selective signatures at genome level has experienced a major breakthrough. Varieties of methods are available to detect genome-wide selective signature using the DNA sequence data and were generally classified by Qanbari [4] in two major categories: intra-population statistics [5, 6] and inter-populations statistics [7, 8]. Intra-population statistics can be split up into two sets based on single site or haplotype linkage disequilibrium analyses, respectively [4]. And of those, Wright’s fixation index, namely FST, which was based on single site differentiation statistic, was applied with the high frequency [9]. On the other hand, the integrated Haplotype Homozygosity Score (iHS) and the extended haplotype homozygosity (EHH) test [5,10] depended on haplotype linkage disequilibrium have been performed in different researches for detecting mutations under positive selection which showed a strong signatures[11, 12].

So far, the EHH test has been applied to detect selective signatures of different animals in many researches [13,14,15,16], and proved to be particularly effective among the various statistics [17,18]. Measuring the characteristics of haplotypes, it could identify selection signatures. To be specific, during the natural selection, the neutral mutation happened with a normal frequency, but quite different in the artificial selection, which showed a more rapid increased and decreased mutation frequency, so that a longer surrounding conserved haplotype could be detected. However, as recombination rates are not always homogeneous among chromosomal regions, the method, like EHH test, just relied on haplotypes may potentially lead to false positives [13]. Therefore, Sabeti et al. proposed REHH (Relative EHH) test, which successfully corrected diverse recombination rates resulting from local variation by comparing the EHH on the trained core haplotype with that of the grouped set of core haplotypes at the region except the trained core haplotype. Above all, the REHH test, which was designed to deal with SNP but not sequencing data, promoted the accuracy of exploring selective signatures. [11, 13].

In the present study, we implemented a genome-wide detection of selective signatures using the EHH test with 600 K Affymetrix Chicken SNP array in an F2 population including 1,534 hens. It is a highlight that the high-density SNP chip has been used to detect selection signatures in a large-scale population. Furthermore, our previous GWAS results were incorporated with these detected core regions, what lead to a better understanding of some important biologic processes and causal variants related to crucial economic traits in chicken.

## Materials and Methods

### Ethical Statement

All the blood samples were collected from brachial veins of chickens by standard venipuncture and the whole procedures were performed in accordance with the Guidelines for Experimental Animals established by the Ministry of Agriculture of China (Beijing, China). The entire study was approved by the Animal Welfare Committee of China Agricultural University (permit number: SYXK 2007–0023).

### Resource Population and Genotyping

An F2 resource population was derived from reciprocal crossed between a popular commercial breed White Leghorn (WL) and a Chinese indigenous strain Dongxiang chickens (DX). We chose 1,534 hens as experimental samples, which was originating from 49 half-sib families and 550 full-sib families. And all the blood samples were collected from brachial veins of chickens by standard venipuncture. Genomic DNA was extracted by standard phenol-chloroform method and the eligible samples were genotyped with the 600 K Affymetrix Axiom Chicken Genotyping Array (Affymetrix, Inc. Santa Clara, CA, USA). All hens were caged individually and reared with feed and water ad libitum in the same environment (Farm of Jiangsu Institute of Poultry Science).

Before analyses, we first removed 7,883 SNPs with unknown chromosome and 112 SNPs with redundant genomic positions. Then we performed the quality control with Affymetrix Power Tools (APT) and R scripts according to the guidelines by Affymetrix (http://affymetrix.com/). We kept the samples with dish quality control (DQC) > 0.82 and sample call rate >99% or SNP call rate >97%, only 532,299 SNPs remained for the following analyses. In addition, we deleted 26,656 SNPs on sex chromosomes and 302 SNPs on the two linkage groups. SNPs with minor allele frequency (MAF) less than 5% and those deviating from Hardy-Weinberg equilibrium (HWE) test (P-value < 1×10^−6^) using the PLINK package [19] were excluded. Besides, we used Beagle V.4.1 [20] to impute some sporadic missing genotypes and to reconstruct haplotypes for every chromosome with the default parameters.

### Genome-wide Detection of Selection Signature

We firstly identified core regions, which were characterized by SNPs with strong linkage disequilibrium (LD) and consisted of some core haplotypes after the EHH test by the software Sweep v.1.1 (http://www.broadinstitute.org/mpg/sweep/) [21] in the whole chicken genome, in which 3 to 20 SNPs located. The plot of LD decay was available in S1 Fig. As the ICGSC (2004) reported, the recombination rate ranged from 2.5 to 21 cM/Mb among the chicken chromosomes [22,23]. And rates were much higher on microchromosomes than on macrochromosomes, where the median value were 6.6 cM/Mb and 2.8 cM/Mb, respectively. Considering that macrochromosomes occupied a large proportion in chicken, physical distance of 300Kb was chosen as the matched distance to determine the REHH value for each core region, as well as evaluating how LD decayed across the whole genome. The REHH (Relative EHH) statistic corrected EHH through eliminating the influence of variability in chicken recombination rates [13].

$$REHH=EHH/\bar{EHH}$$

Which the $\bar{EHH}$ was defined as the decay of the special core haplotype of EHH on all other core haplotypes.

Furthermore, we treated and ordered the frequency of all core haplotypes into 20 bins to compute the significance of the REHH values, obtaining P-values by log-transforming to reach normality and calculating the mean and standard deviation.

### Annotation and Functional Analysis

After performing EHH tests, regions with extreme REHH p-values were considered as candidates for selective sweeps, as proposed by Sabeti et al.[5]. Comparing with chicken QTL database (http://www.animalgenome.org/cgi-bin/QTLdb/GG/index)[24], we firstly screened the distribution of the candidate selective signature regions located in QTL using the Perl script. Genes participated in the significant core regions were annotated using the online Genome Browser and Biomart tools by Ensembl [25, 26]. We also compared these genes with what found in our previous GWAS, in order to dig interesting genes determining or affecting some important economic traits. Functional analyses were carried out for the sweeps identified in the F_2_ population using the function annotation and clustering tools in the Database for annotation, Visualization and Integrated Discovery (DAVID) [27].

## Results

### Descriptive Statistics for Markers and Core Haplotype

With an average neighbor marker distance of 1.79 kb, 580,961 SNPs were genotyped by using 600 K Affymetrix Axiom Chicken Genotyping Array. After the quality control and discarding the SNPs on two linkage groups and sex chromosome, 389,618 SNPs and 1,512 individuals were finally remained for the further analyses. Table 1 completely described the distribution of SNPs and haplotypes identified among the whole chicken genome.

**Table 1:** Summary of genome-wide marker and core region (CR) distribution in the F2 population.

Chr	Chr length (Mbp)	dbSNP (n)	Mean Distance(Kb)	No.CR (n)	Coverage CR length(Mbp)	Mean CR length(Kb)	Max CR length(Kb)	CR length / Chr length(%)	CR SNP (n)	Mean CR SNP (n)	CR SNP/ dbSNP (%)
1	195.28	102351	1.91	8988	105.54	11.74±14.26	181.22	54.05	50543	5.62±4.00	49.38
2	148.81	64435	2.31	5818	75.73	13.02±15.42	248.10	50.89	31677	5.44±3.98	49.16
3	110.45	57233	1.93	5313	59.87	11.27±12.02	99.71	54.21	29376	5.53±3.89	51.33
4	90.22	43337	2.08	4009	46.36	11.56±12.64	212.57	51.39	21835	5.45±3.82	50.38
5	59.58	30617	1.95	2802	30.78	10.98±21.20	965.16	51.66	15072	5.38±3.65	49.23
6	34.95	21943	1.59	2013	18.13	9.00±10.11	161.71	51.87	10786	5.36±3.62	49.15
7	36.24	21604	1.68	1963	19.18	9.77±13.90	267.44	52.92	10831	5.52±3.78	50.13
8	28.77	17274	1.67	1495	16.84	11.27±13.09	127.78	58.53	9043	6.05±4.46	52.35
9	23.44	18117	1.29	1677	11.12	6.63±6.46	56.73	47.44	8412	5.02±3.22	46.43
10	19.91	18947	1.05	1761	10.47	5.95±7.01	171.50	52.59	9388	5.33±3.54	49.55
11	19.40	13984	1.39	1286	10.53	8.18±9.78	100.73	54.28	7089	5.51±3.84	50.69
12	19.90	14829	1.34	1342	9.68	7.21±7.55	71.92	48.64	6817	5.08±3.17	45.97
13	17.76	11282	1.57	1060	8.39	7.91±8.41	97.41	47.24	5189	4.90±2.95	45.99
14	15.16	13181	1.15	1180	7.65	6.48±7.65	104.43	50.46	6128	5.19±3.38	46.49
15	12.66	10505	1.21	970	6.41	6.61±7.83	110.57	50.63	5144	5.30±3.51	48.97
16	0.54	584	0.92	33	0.11	3.23±3.88	18.77	20.55	145	4.39±2.15	24.83
17	10.45	9379	1.11	860	4.63	5.38±6.70	83.10	44.31	4070	4.73±2.98	43.39
18	11.22	9673	1.16	869	4.95	5.69±13.43	313.99	44.12	4086	4.70±2.71	42.24
19	9.98	9044	1.10	790	5.28	6.68±8.19	75.77	52.91	4395	5.56±3.76	48.60
20	14.30	9614	1.49	864	7.13	8.25±11.23	169.91	49.86	4527	5.24±3.23	47.09
21	6.80	8943	0.76	809	3.52	4.35±6.40	122.99	51.76	4287	5.30±3.25	47.94
22	4.08	4696	0.87	361	2.68	7.43±16.16	138.43	65.69	2238	6.20±4.33	47.66
23	5.72	6687	0.86	613	2.77	4.52±6.67	80.71	48.43	2896	4.72±2.66	43.31
24	6.32	7745	0.82	667	3.25	4.88±5.47	74.36	51.42	3559	5.34±3.53	45.95
25	2.19	2501	0.88	185	0.8	4.31±4.68	41.29	36.53	1000	5.41±3.19	39.98
26	5.33	6332	0.84	504	2.39	4.75±6.30	70.12	44.84	2554	5.07±3.23	40.33
27	5.21	5731	0.91	460	2.89	6.28±22.26	306.67	55.47	2237	4.86±2.80	39.03
28	4.74	5553	0.85	459	2.45	5.34±9.49	133.39	51.69	2516	5.48±3.58	45.31
Total	919.41	546121	1.31	49151	479.51	9.76±13.11	965.16	52.15	265840	5.41±3.73	48.68

**Fig 1:**
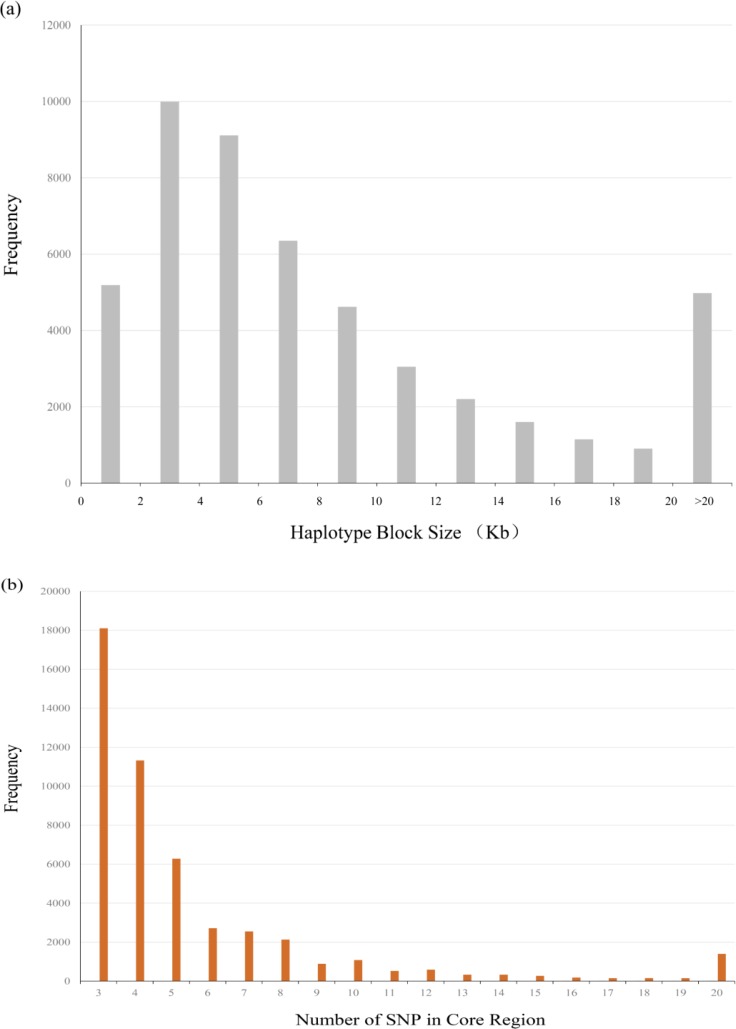
The distribution of the size of haplotypes and the number of SNPs in the core regions (a) and (b).

For the SNPs analyzed in this study, a total of 49,151 core regions covering 479.51 Mbp (52.15%) of the genome were uncovered. The average length of core region was approximated as 9.76±13.11 Kb, while the maximum was of 965.16 Kb located in chromosome 5. For the every chromosome, the covered proportion of length by core regions with total length, as same as the number of SNPs, were also given in Table 1. What’s more, we draw Fig 1 to depict the distribution of the size of haplotype blocks as well as the number of SNPs within core regions. Overall, there were 265,840 SNPs (48.68%) located in core regions, and the number of them were sprayed between three to twenty in each core, since twenty was designed as upper limit for the SNPs, even if it may exceed.

### Genome-wide Selection Signature

**Fig 2:**
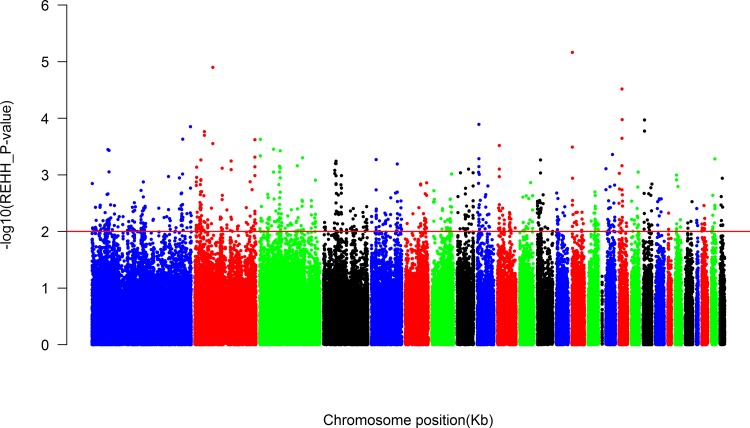
The distribution of the P-values of haplotypes with frequency>0.25 on the whole genome. Continuous red lines display the threshold level of 0.01.

A total of 434,509 EHH tests were performed in 49,151 core regions and averaged out to 8.84 tests per core region. Core haplotypes under selection would have a relatively high frequency according to the selection signature theory. Hence, the core haplotypes with frequency below 25% were totally excluded. The distribution of remaining core haplotypes across the whole genome was visualized via a Manhattan plot in Fig 2, which displayed the P-value of REHH by minus log-transforming located in the different chromosomal position. It was evident that these selection signals mainly concentrated in macro-chromosome such as chromosomes 1, 2, and 3.

**Fig 3:**
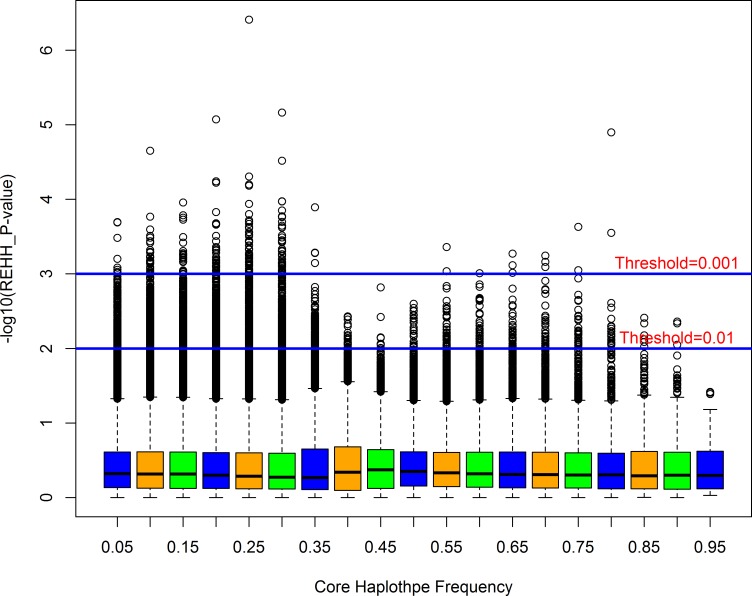
Box plot of the distribution of P-values in core haplotype frequency bins in the F2 population. The two continuous blue lines indicated the threshold P-values of 0.01 and 0.001, respectively.

**Fig 4:**
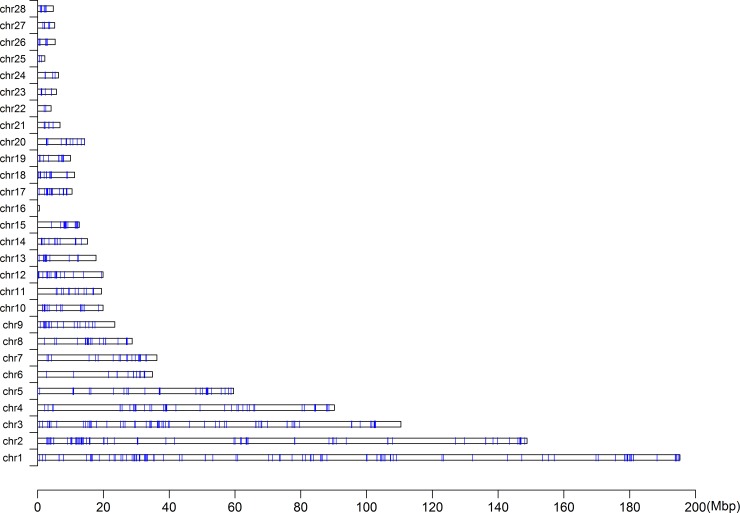
The genome-wide map of selective signature. The blue vertical line represented the selective signature across the genome.

Table 2 indicated that 149,662 EHH tests were remained for all core haplotypes with the frequency > 25%. There were 5,166 and 806 tests reaching significant level with the P-value < 0.05 and 0.01, respectively. We then examined the conformity of the distribution of Tukey’s outliers with the threshold level of the P-value of 0.01 and 0.001 in core haplotypes. The–log_10_ of the REHH P-value distributed within each bin, which was partitioned by core haplotype frequency, was displayed in Fig 3. As it showed that the majority of the extreme outliers appeared with small haplotype frequencies. The genome-wide map of selective signature was shown in Fig 4. The blue vertical line represented selective signature across the whole genome.

**Table 2:** The number of tests on core haplotypes (CH) with frequency > 0.25 and P-values of REHH test.

Chr	Tests on CH	P-value <0.05	P-value <0.01	Chr	Tests on CH	P-value <0.05	P-value <0.01
1	27693	918	142	15	2922	115	22
2	17089	588	99	16	58	0	0
3	16866	658	81	17	2682	85	25
4	12383	366	58	18	2427	71	22
5	8439	302	46	19	2297	86	13
6	6395	220	21	20	2474	75	18
7	6055	210	28	21	2430	87	8
8	4660	174	30	22	1008	28	2
9	4627	135	30	23	1859	88	9
10	5187	164	35	24	2114	79	4
11	3945	134	17	25	522	21	4
12	4323	166	28	26	1661	58	11
13	3327	103	16	27	1401	53	10
14	3494	124	18	28	1324	58	9
Total					149662	5166	806

### Genome Annotation within Significant Regions

Core regions owning the significant P-values (P < 0.01) of REHH were explored to identify all overlaps with published QTLs in the chicken QTL database which was available online. The current release of the Chicken QTLdb contains 4,676 QTLs (319 different traits) from 224 publications. As a consequence, the overlapping core regions were detected to be mainly associated with production traits and immune function, and the lowest P-values (top six) were displayed in S1 Table.

We further annotated overlapping chicken genes located in significant core regions based on Ensembl gene database. A summary of statistics was shown in S2 Fig, and there were 232 genes found within positively selected regions and about half of them were located on chromosomes 1, 2 and 3.

The subset of genes in all core regions with extreme REHH P-values (p < 0.001) were displayed in Table 3. Genes, including *AASDHPPT* (aminoadipate-semialdehyde dehydrogenase-phosphopantetheinyl transferase), *GDPD5* (glycerophosphodiester phosphodiesterase domain containing 5), *PARD3* (par-3 family cell polarity regulator) and *SOX6* (SRY-box 6), were related to immune function and neurogenesis. Particularly, *AKT1* and *GPC1* participating in AKT signal pathway and Wnt signal pathway respectively, which played a key role in organogenesis and reproduction performance, were obtained.

**Table 3:** Summary statistics for genes in extreme significant Core Region (CRs) with P-value<0.001 after the REHH test.

Chr	Core position	Hap fre	REHH_P-value	Genes
1	32948320–32951289	0.29	0.000369	MON2
1	180329883–180332900	0.29	0.000969	AASDHPPT
1	180540921–180545521	0.74	0.000234	GRIA4
1	194101471–194106123	0.29	0.000141	GDPD5
2	3845018–3859990	0.29	0.000733	NBEAL2
2	13436842–13448595	0.27	0.000546	PARD3
2	21228144–21238873	0.26	0.000173	ZNF804B
2	90066439–90067638	0.65	0.000569	LRRC16A
2	146714090–146731491	0.29	0.000239	TSNARE1
2	146874393–146882078	0.29	0.000722	TSNARE1
3	37837568–37839943	0.27	0.000376	SLC35F3
5	10760242–10762655	0.62	0.000536	SOX6
5	51535169–51545347	0.27	0.000645	AKT1
8	19982263–19997665	0.29	0.000792	TESK2
9	2223866–2226685	0.33	0.000716	GPC1
10	2100409–2112553	0.29	0.000304	ISLR2
12	5270938–5278515	0.30	0.000547	IQSEC1
17	6626503–6628357	0.54	0.000437	DDX31

### Functional Enrichment Analyses

The annotations of genes and analysis of pathways were conducted using online DAVID software [27]. The genes were found to be significantly (P<0.05) enriched in 10 Go terms (Table 4). The term of ‘cell part morphogenesis’ (GO: 0032990) indicates genes associated with immune function. The term of ‘eye development’ (GO: 0001654) and ‘neuron projection morphogenesis’ (GO: 0048812) suggested the distinct biological association with organ development and neurogenesis in chicken.

**Table 4:** Enrichment of Gene Ontology (GO) among the positively selected regions.

Term(Biological process)	Gene number	P-Value
GO:0032990	5	0.015
cell part morphogenesis		
GO:0006357	8	0.02
regulation of transcription from RNA polymerase II promoter		
GO:0022610	9	0.023
biological adhesion		
GO:0007155	9	0.023
cell adhesion		
GO:0007638	2	0.04
mechanosensory behavior		
GO:0001654	4	0.041
eye development		
GO:0007409	4	0.043
axonogenesis		
GO:0048812	4	0.046
neuron projection morphogenesis		
GO:0030030	5	0.046
cell projection organization		
GO:0007423	5	0.048
sensory organ development		

## Discussion

Chicken are reported to have gone through strict artificial selection and breeding for multi-purpose as far as thousands years ago [28] and can be served as an experimental model in studying important biological process and effective disease treatment [29, 30, 31]. Especially the improvement of the chicken genome sequence (ICGSC Gallus_gallus-4.0/galGal4 Nov.2011) makes it possible to reveal the genetic basis under chickens’ recent evolution across the whole genome. To be specific, identifying the genes in the core regions that have experienced artificial selection would effectively explicate economic traits or biological process due to breeding. For example, Zhang et al. [32] genotyped 475 DNA samples of two divergent chicken lines that were selected for abdominal fat (AF) content. They detected 51 and 57 significant core regions in the lean and fat lines, respectively, with some important genes involved in AF deposition in chickens. In present study, EHH test was conducted to identify the selection signatures across the whole genome and bioinformatics analyses was applied to convince the biological significance for the detected core regions in F_2_ chicken population. Furthermore, we took fully advantage of high density SNP chips which contain much more information, so that to improve the accuracy of detection.

The 806 significant core regions (S2 Table) identified in this study was much more than previous report because of the high density SNP chip and genome recombination events [33, 34]. We further compared significant core haplotypes with other haplotypes in these regions and found that the former possess the larger extent of homozygosity than the later. Therefore, we inferred that the long stretch of homozygosity were not only simply resulted from the strong selective pressure but also owed to the local recombination rate [35]. Given that the method of EHH test may failed to detect the lower-frequency alleles because of the lack of sensitivity, [36], we removed the haplotypes with frequency below 25%. It would contribute to reduce the frequency of false positives as far as possible and lead to an authentic result. We then aligned significant core regions (*P* < 0.01) with the chicken QTL database. These positively selected regions were illustrated to be relevant to some important economic traits, like egg production, feed efficiency, body weight and immunity. These results were in accordance with the findings reported by Li DF et al. [14].

To elaborate whether the selection signatures were correlative with phenotypic traits, we compared them with our previous genome-wide association studies. In our previous GWAS studies [37, 38, 39], 37 genes played a key role in egg production and feed efficiency in chicken such as egg weight (EW), egg number (EN), feed intake (FI), feed conversion ratio (FCR) and residual feed intake(RFI). Twenty-two genes overlapped with core regions had been demonstrated to be associated with economic traits. For instance, the *NCAPG* gene locating on chromosome 4 had been identified influencing both egg weight and body weight simultaneously in a pleiotropic manner [37]; the *CLSPN* gene on chromosome 23 displayed significant haplotype [38], may related with egg number via affecting the function of ovary and uterus[40,41]. These results could effectively elucidate that some important economic traits like egg production had undergone selection.

As shown in S2 Fig, a total of 232 genes were screened within positively selected regions and most of them were located on macrochromosomes due to the greater length of themselves. Furthermore, a panel of genes emerging in the extreme significant core regions with P-value of REHH < 0.001 including *AASDHPPT*, *GDPD5*, *PARD3*, *SOX6*, *AKT1* and *GPC1*, were testified to be able to exert influence on some biological process. Among them, the *AASDHPPT* gene was reported to have a role in either the adaptive or innate immune response [42]. The *GDPD5* gene, sharing homology with glycerophosphodiester phosphodiesterase 2 (*GDE2*), is important to initiate neurogenesis and cellular proliferation and differentiation [43]. According to Afonso’s study [44], the morphogenesis of embryonic neural tissue and the process of neurogenesis in chicken were partly regulated by *PARD3*. Additionally, the positive feedback loop between *Sox2* and *Sox6*, whose functions were to inhibit premature neuronal differentiation, played a key role in maintaining the neural progenitor cells [45]. The other genes were involved in two critical signaling pathway, namely PI3K/Akt pathway and Wnt signal pathway. The PI3K-Akt pathway participated in early infection of some exogenous avian leucosis viruses [46] and could mediate IGF-1 survival during the otic neuronal progenitor phase of early inner ear development [47]. The *GPC1* gene involved in Wnt pathway was of great importance in chick, which can regulate the signaling mechanisms in early formation of the trigeminal sensory system and cell proliferation hence affected reproduction performance [48,49].

The significant GO terms were shown in table and the terms of biological process appealed to us mostly. The terms including ‘cell part morphogenesis’, ‘eye development’, ‘neuron projection morphogenesis’ and ‘sensory organ development’ were consistent with our previous result about the function of the extreme significant genes. Among our findings, genes participated in these terms that overlapping with positive selection core regions, like *VSX2* and *Bmp7*, which were associated with cell growth [50] and sensory organ development [51,52], played a key role in chicken. Unfortunately, no pathway achieved significant level (P-value < 0.05). The explanation may be able to account for the result was that the current annotation of the chicken genome limited availability of genes mapped in the KEGG pathways, further decreased the sensitivity of the analysis. Hence, it’s in urgent need of new efficient technique to enhance the efficiency for the detection of selection signatures in different populations in the future. It might be helpful to combine the diverse test in a composite likelihood statistic, because each single test only provided partial information of selective signatures [36].

In conclusion, 806 significant core regions were detected across the whole genome in chicken applying the EHH test together with certain bioinformatics analyses. Genes in these regions related to immune function, sensory organ development and neurogenesis may experience positive selection. Moreover, our results draw a comparatively integrated genome-wide map of selection signature in chicken and yielded valuable insight into the genetic basic of artificial selection in poultry breeding.

## Supporting Information

S1 Fig Decay of linkage disequilibrium with distance on the whole genome. (TIFF)

S2 Fig The distribution of all genes within positively selected regions. (TIF)

S1 Table Traits and the position of the overlapping chicken QTL for the core regions with lowest P-values (top six). Including traits information, QTL and the QTL IDs.
(XLSX)

S2 Table Candidate selected regions positions in this population. Including the chromosome number, start position, end position, length and REHH P-value.
(XLSX)
