# Supplementary figures and images for: Genome-Wide Detection of Selective Signatures in Chicken through High Density SNPs
Source: PLoS One. 2016 Nov 7;11(11):e0166146. doi: 10.1371/journal.pone.0166146 (PMC5098818; doi:10.1371/journal.pone.0166146)

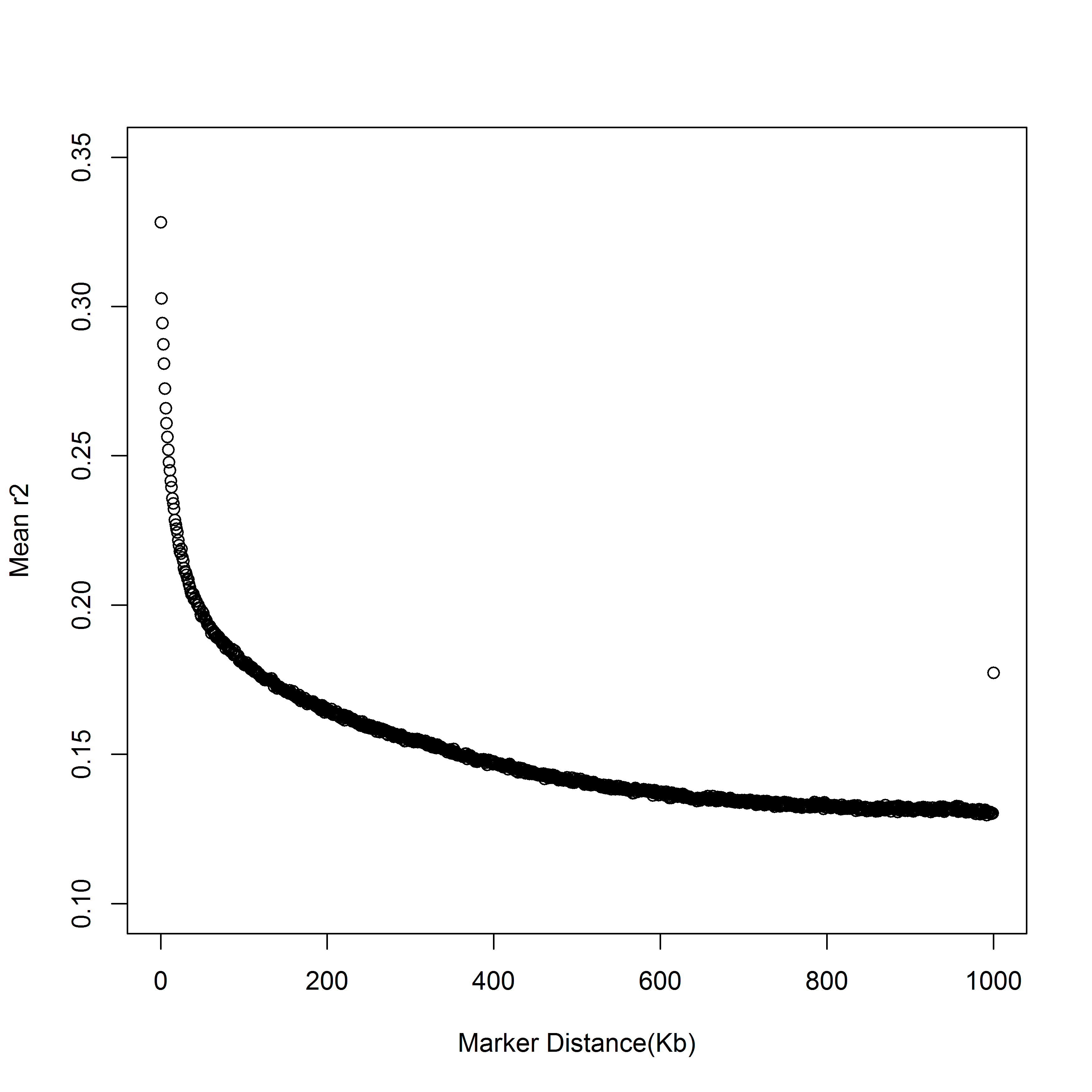

Supplement: S1 Fig — (TIFF) [file pone.0166146.s001.tiff]

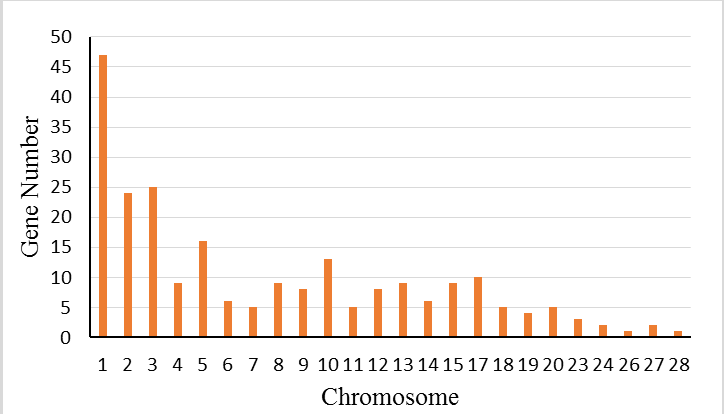

Supplement: S2 Fig — (TIF) [file pone.0166146.s002.tif]
